# Regulatory frameworks can facilitate or hinder the potential for genome editing to contribute to sustainable agricultural development

**DOI:** 10.3389/fbioe.2022.959236

**Published:** 2022-09-30

**Authors:** Hellen Mbaya, Simon Lillico, Steve Kemp, Geoff Simm, Alan Raybould

**Affiliations:** ^1^ Global Academy of Agriculture and Food Systems, The Royal (Dick) School of Veterinary Studies and The Roslin Institute, University of Edinburgh, Midlothian, United Kingdom; ^2^ Centre for Tropical Livestock Genetics and Health (CTLGH), Roslin Institute, University of Edinburgh, Edinburgh, United Kingdom; ^3^ Centre for Tropical Livestock Genetics and Health (CTLGH), ILRI Kenya, Nairobi, Kenya; ^4^ Innogen Institute, Science Technology and Innovation Studies Department, University of Edinburgh, Edinburgh, United Kingdom

**Keywords:** genome editing, genetic modification, new breeding techniques, CRISPR, regulatory frameworks

## Abstract

The advent of new breeding techniques (NBTs), in particular genome editing (GEd), has provided more accurate and precise ways to introduce targeted changes in the genome of both plants and animals. This has resulted in the use of the technology by a wider variety of stakeholders for different applications in comparison to transgenesis. Regulators in different parts of the world are now examining their current frameworks to assess their applicability to these NBTs and their products. We looked at how countries selected from a sample of geographical regions globally are currently handling applications involving GEd organisms and what they foresee as opportunities and potential challenges to acceptance of the technology in their jurisdictions. In addition to regulatory frameworks that create an enabling environment for these NBTs, acceptance of the products by the public is vitally important. We, therefore, suggest that early stakeholder engagement and communication to the public be emphasized to foster public acceptance even before products are ready for market. Furthermore, global cooperation and consensus on issues cutting across regions will be crucial in avoiding regulatory-related bottlenecks that affect global trade and agriculture.

## 1 Introduction

In their report on The State of Food and Agriculture, 2021, the Food and Agricultural Organization of the United Nations (FAO) defined sustainable development as the management of economic, social, and environmental resources and technological and institutional changes to attain and continue to meet the human needs of present and future generations (FAO, 2021). Food supply chains and the livelihoods of agri-food systems’ actors are increasingly disrupted by both short-term disturbances—from droughts and floods to armed conflict and food price hikes—and long-term stresses, including climate change and environmental degradation (FAO, 2021).

Climate change poses a severe threat to the future of the environment as it pertains to nearly every aspect of our world including agriculture, biodiversity, and functions of the human society. In response to the challenges of climate change, genome editing (GEd) has been identified as one of the tools that can be applied to either facilitate the adaptation of organisms to climate change or help in the mitigation of the effects of climate change on agriculture (Karavolias et al., 2021).

### 1.1 History of adoption of GMOs and emergence of NBTs

Current regulatory regimes for GM crops are either product-based, as is seen in the United States, Argentina*,* and Canada, or process-based as seen in the EU and Australia (Hartung, 2014; Ishii and Araki, 2017). Product-based regulatory regimes focus on whether the final product has a “novel combination of genetic material,” while process-based systems focus on the technology or process applied to give rise to the final product. With respect to our article, genetic modification refers to a technique whose aim is to change the characteristics of a plant, animal, or microorganism by transferring genes from one organism to an organism of a different species (transgenesis) or of the same species (cisgenesis) This is performed through targeted isolation of the desired genes from the DNA of one organism and adding them to the other organism. In contrast, genome editing encompasses a group of technologies that give scientists the ability to change an organism’s DNA by adding, removing, or altering genetic material at particular locations in the genome (Ran et al., 2013).

In the 22 years up to 2017, an accumulated 2.34 billion hectares of GM crops were grown commercially worldwide, comprising 1.13 billion hectares of GM soybean, 0.7 billion hectares of GM maize, 0.36 billion hectares of GM cotton, and 0.14 billion hectares of GM canola. Products derived from these crops significantly contribute to food, feed, fiber, and fuel for the current world population of almost 7.7 billion people (ISAAA, 2019). Nevertheless, only 26 countries globally account for this output of GM crops, and in particular, Brazil and the US accounted for 65% of the worldwide area of GM crops in 2019. This has been attributed to regulatory asymmetry and the polarized debate about GM crops and animals, which has resulted in slow adoption and public acceptance in many parts of the world. Additionally, the cost, time, and dedicated capacity required to satisfy regulatory requirements have resulted in regulation acting as a barrier to entry for smaller traditional seed-producing firms (Tait, 2007).

Klümper and Qaim (2014) showed that the yield benefits of GM crops are significantly higher than their non-GM counterparts. Despite this, the latter, especially if grown with organic practices, are often regarded as more sustainable, and technologies such as GM and GEd are often ignored by policymakers and their advisors in discussions about sustainability. In a 2019 report titled Net Zero—The UK’s contribution to stopping global warming (Climate Change Committee, 2019), for example, the UK’s Climate Change Committee makes no reference to biotechnology or GM crops, even though agriculture, farm, or farming are mentioned 135 times and technology is mentioned 79 times. However, the concept of sustainability has recently begun to be considered a key point for public opinion. For example, the European Commission initiated a policy action to assess the impact of different legislative options regarding GEd products, which includes a public consultation that touches on the issue of sustainability.

Failures by policymakers to consider GM and GEd products for sustainable development are possibly due to the association of the former with industrial agriculture and a near monopoly by multinationals. In addition, campaigns by international environmental non-governmental organizations (NGOs) against the technology have contributed to negative perceptions and a lack of public acceptance of GM products (The Guardian, 2016; Paarlberg, 2014). However, recent studies have examined the use of GEd technologies as one of the contributors to sustainable development and mitigation of the effects of climate change (Bierbaum et al., 2020; Karavolias et al., 2021). Sharing such studies with the public and making policymakers more aware of the potential of biotechnology for sustainable development may lead to a change in perceptions of the use of technologies to improve current crops and livestock, as well as aid in the fight against climate change.

An increase in the availability of agricultural biotechnologies in both animal and plant breeding has forced governments around the world to adjust, or completely overhaul, their regulatory regimes to either embrace or forgo the benefits of these technologies (Lassoued et al., 2021). GEd comprises powerful new techniques that could distribute innovation in agricultural biotechnology among a wider set of product developers and have more direct benefits to consumers (Lema, 2019). To realize these opportunities, any specific regulation of GEd needs to encourage innovation and ensure that the risks from using GEd products are acceptable (Raybould, 2021). Such an outcome is unlikely to be achieved simply by the wholesale application of current GM regulations to GEd products.

As with unintended effects of genetic modification, concerns have been raised about unintended genetic changes that may be caused by off-target effects of gene editing (Zhao and Wolt, 2017). However, a developing consensus among experts is that unintended changes introduced by GEd are no more likely to be harmful than the many unintended changes introduced by other methods of plant breeding (Lassoued et al., 2021). In addition, the risks of unintended changes caused by genetic modification seem to have been overstated (Smyth et al., 2021). Additionally, with the huge development in the knowledge of genomes and in the sequencing potential, there are tools to thoroughly detect the occurrence of any off-target modifications.

Some authors advocate for wider stakeholder engagement in the decisions about the development of regulations for these organisms, for example, concerns about the welfare of gene-edited animals (Bruce, 2017) and consumers’ desire for traceability of products derived from GEd organisms (Ortega et al., 2022) are topics that may influence decisions about whether to regulate GEd organisms. In order to assist countries to make decisions on how to regulate GEd organisms and/or their products, we conducted a study to

i. review the current regulatory practices for GEd organisms in selected countries;
ii. identify the challenges and opportunities of GEd organisms in these countries; and
iii. identify other factors critical in creating an enabling environment for the uptake of new technologies such as GEd.

## 2 Methods

A questionnaire was designed for interviews with officers in regulatory agencies and academics from selected countries. The countries selected have regulations in place for GM crops and animals, have commercialized or grown GM crops, and/or trade in GM products. Additionally, interview participants were selected from different geographical regions comprising North America (the US and Canada), South America (Argentina and Brazil), Asia (China and Japan), Africa (Kenya and Zambia), the EU, and finally the Pacific region (Australia). A copy of the interview questions is available as supplementary information. The questionnaire was submitted for ethical approval at the University of Edinburgh, Royal (Dick) School of Veterinary Studies’ Human Ethics Research Committee (HERC) and was granted approval in June 2020. Thereafter, interviews took place between August 2020 and March 2021. Some of the interviews were conducted by email, while others through online video platforms (Blackboard Collaborate). The questions comprised both open-ended and multiple choice questions whose responses were recorded and transcribed after the interviews. The interview questionnaire was designed with the aim of understanding the day-to-day experience with GEd applications from the regulators and academicians who had the experience of submitting an application in their respective countries.

## 3 Results

### 3.1 How do countries currently regulate agricultural biotechnologies?

Table 1 gives a summary of the current approaches to both GM and GEd technology regulation by the countries selected for the regulatory interviews. Briefly, in Australia and Japan, current guidelines were amended to include NBTs such as GEd; in Argentina, the United States, and Zambia, GEd applications are handled on a case-by-case basis, while for Brazil, Kenya, and China, new regulations were developed to cater to new breeding techniques. In the case of Canada, the novelty of the trait in the final product for GM or GEd technologies is used to determine the regulatory approach, while the EU currently regulates GEd products using the same framework previously developed for GM products.

**TABLE 1:** Summary of current country approaches to both GM and GEd technology regulation.

Country	GM regulatory trigger	GEd regulation approach
Argentina	Product-based	Gene editing applications are handled on a case-by-case basis
Australia	Process-based	Current regulations were amended to include new breeding techniques
Brazil	Product- and process-based	New regulations were developed to cater to new breeding techniques, for example, gene editing
Canada	Product-based	No new regulations were developed, and GE organisms are subjected to the same oversight as their conventional counterparts
China	Process-based	New regulations were developed to cater to new breeding techniques, for example, gene editing
EU	Process-based	**Current regulations were amended to include new breeding techniques—**no amendment, but ECJ ruling clarified how existing regulation has to be interpreted so as to include GEd organisms into the legal framework
Japan	Product- and process-based	Japanese government issued several guidance documents in 2019 to the public on how to interpret existing GM regulations and whether provisions of these regulations are applicable (or not) to gene-edited products
Kenya	Product- and process-based	New regulations were developed to cater to new breeding techniques, for example, gene editing
United States	Product-based	Depends on the agency and situation
		Food and Drug Administration (FDA)/Center for Veterinary Medicine (CVM): current regulations were amended to include new breeding techniques
		Environmental Protection Agency (EPA) and FDA: gene editing applications are handled on a case-by-case basis
		United States Department of Agriculture (USDA): new regulations were developed to cater to new breeding techniques, for example, gene editing
Zambia	Process-based	Gene editing applications are handled on a case-by-case basis

### 3.2 GEd applications submitted to selected regulatory authorities and their contribution toward sustainability

A summary of GEd applications submitted to regulatory authorities in the selected countries is given in Table 2. Based on the responses received for our interview questionnaire, it is evident that GEd techniques are being applied to a wide variety of food crops and livestock, with experimental yield increases showing significant potential for GEd crops to contribute to reducing food insecurity, as well as combating the effects of changing climates, as reported by Smyth et al., 2021.

**TABLE 2:** Summary of the GEd applications improving sustainability and nutritional value traits, submitted to selected regulatory authorities.

Country	Organism	Trait targeted in GEd application submitted
Australia	Cattle	•Heat tolerance
		•Hornless dairy
	Coral reef	•Heat-tolerant select species
	Potatoes	•Low-glycemic index
	Chicken	•Reduced allergenicity in chicken eggs
		•Single sex—female only
	Carp	•Population control
*Argentina*	Potatoes	•Non-browning
	Cattle	•Reduced allergenicity in milk
Brazil	Corn	•Extra starch
	Yeast	•Bioethanol
	Cattle	•Hornless
China	Tomato	•Heat tolerance
	Pigs	•Tolerance to cold temperatures and leaner meat
	Cattle	•Tuberculosis-resistant
EU	*Camelina*	•High oleic acid
	Potato	•Stable starch
Japan	Wheat	•Rain-resistant
	Tomato	•Gamma-aminobutyric acid (GABA)-enhanced
	Potato	•Low starch
Kenya	Yam	•Disease resistance and enhanced vitamin A
	Sorghum	•Resistance against Striga (*Striga hermonthica*)
United States	Soybean	•Altered oil content
	Cattle	•Heat tolerance

From the summary given in Table 2, applications that are targeting climate resilience comprised heat-tolerant cattle and coral reef conservation in Australia, heat-tolerant tomatoes and pigs that can withstand cold temperatures in China, and finally rain-resistant wheat in Japan. Furthermore, those that were modified for nutritional enhancement comprised chicken eggs and milk with reduced allergenicity in Australia and Argentina, respectively. Applications submitted also targeted disease resistance as in the examples of tuberculosis-resistant cows from China, disease-resistant yam, and Striga-resistant sorghum from Kenya (weed resistance).

### 3.3 GEd applications submitted to selected regulatory authorities and their contribution toward yield improvement

Interview respondents from Argentina, China, the EU, and Japan reported having received applications submitted for yield improvement, which are summarized in Table 3. They comprise alfalfa from Argentina targeting productivity and improved quality, rice and maize from China targeting yield increase, and with respect to livestock, pigs targeted for fast growth, more muscle, and less fat, and goats targeted for more muscle.

**TABLE 3:** Summary of applications involving GEd crops and animals for yield improvement.

Country	Organism	Trait targeted in GEd application submitted
*Argentina*	Alfalfa	•Higher quality
China	Rice	•High yield
	Maize	•High yield
	Pigs	•Fast growing
		•More muscle
		•Less fat
	Goats	•More muscle
Japan	Tiger puffer fish	•Fast growing
	Sea bream	•More muscle

## 4 Opportunities, challenges, and other factors key for uptake of genome editing

In the subsequent sections, the opportunities and challenges with respect to GEd are discussed, based on interview and questionnaire responses from the regulatory officers and academics. Briefly, the respondents identified two main opportunities associated with GEd technology, the potential contribution to sustainable agriculture and improved access to innovation. The challenges for the technology include potential trade issues due to regulatory asymmetry, public perception, and the potential for detrimental effects. In conclusion, other factors listed as key aspects to uptake of genome editing include predictability/legal certainty, end-user empowerment, and global cooperation.

### 4.1 Opportunities presented by GEd technology

#### 4.1.1 Contribution to sustainable agriculture

Given their broad range of potential applications, NBTs such as GEd offer flexible and affordable tools to achieve growth in agricultural productivity with simultaneous improvements in sustainability, natural resource management, equity, food affordability, and farm profitability. There is also a growing recognition of the role that GEd products can play in helping to address global challenges such as improving food security and nutrition, mitigating and adapting to climate change, and responding to pest and disease pressures. Additionally, technological advances such as GEd in animals offer exciting promise for the development of products that address public health concerns such as disease transmission. This would reduce the use of antimicrobials and consequently reduce selection pressure for antimicrobial resistance (AMR) in the process and contribute to a sustainable food supply.

#### 4.1.2 Equitable access to technology

Equitable access to new technologies is an important issue for NBTs and, in particular, for smaller developers and public sector institutions. For example, the USDA respondent shared that the US animal livestock sector is concerned that they will not have access to traits made possible by GEd, and they are further concerned that farmers in other countries will have access to these traits, placing them at a competitive disadvantage.

In countries with favorable regulatory regimes, opportunities have arisen that have led to accelerated breeding and wider opportunities for small- and medium-sized seed companies, as well as opportunities for a university/public research institute to get involved in GEd business (Lema, 2019). Furthermore, newer approaches by USDA, as demonstrated with Am I Regulated (AIR) inquiries, have resulted in an increase in developments by smaller developers and public research institutions. The types of crops that are being developed have broadened both with regard to types of plants (for example, more specialty crops) and the types of traits being introduced (USDA). These are just some examples where GEd has improved access to technological innovation by small- and medium-sized stakeholders in comparison to GM technology where only multinationals could afford to invest because of the huge regulatory costs and onerous processes involved.

### 4.2 Challenges foreseen in the adoption of GEd technology

#### 4.2.1 International trade due to regulatory asymmetry

A potential challenge foreseen to result from the adoption of GEd crops and animals is consumers’ concern over GEd food and the risk for exporters who export the products unintentionally to countries that have different regulatory schemes from their own. This emphasizes the importance of an international discussion about the regulation of GEd to avoid the current challenges experienced by GM crops and animals. As an example, part of value chain stewardship in the Canadian industry typically voluntarily delays the commercialization of products of biotechnology until required approvals have been obtained in all major markets. As a result, without concerted efforts to minimize the impacts of regulatory asymmetry and asynchrony, products made from GEd could be subject to an unpredictable trade environment that could impede their commercialization.

#### 4.2.2 Public perception and acceptance

Public perception and acceptance continue to be a challenge, and these were mentioned by a majority of the interview questionnaire respondents. This will be even more challenging as products that in most parts of the world are not produced *via* biotechnology are introduced (e.g., specialty crops and animals). In some countries, there are also concerns about potential economic impacts on the organic and GM-free-food sector.

#### 4.2.3 Potential detrimental effects

As with any new technology, there is concern about the potential risks associated with the use of GEd. It can be argued that every food item whether conventional, transgenic, or GEd carries with it a certain degree of risk and that this can vary depending on subsequent processing prior to consumption. Some products developed using GEd are novel, and it will be necessary for a country’s regulatory authorities to determine the acceptable level of risk. Possibly, an international framework for risk assessment of GEd products can be developed that countries could then modify to suit their local circumstances.

### 4.3 Factors critical to the realization of GEd potential for sustainable agricultural development

Many factors contribute to the success or failure of innovations**.** In addition to regulations, respondents were asked to give factors they believed to be crucial to the uptake of new technologies in each of their respective regions. These factors included predictability, end-user empowerment, and global cooperation.

#### 4.3.1 Predictability/legal certainty

Establishing legal certainty as to the regulatory status of new technologies was listed as a priority by the Office of the Gene Technology Regulator in Australia. This provides certainty to product developers considering their path to market and provides assurance to the Australian public that any risks to human health and safety and the environment will be appropriately managed.

In the US, the FDA encourages developers of innovative products to utilize their voluntary consultation process. Additionally, the FDA/CVM has established the Veterinary Innovation Program (VIP) intended to facilitate advancements in the development of innovative animal biotechnology products by providing greater certainty in the regulatory process, encouraging development and research, and supporting an efficient and predictable pathway to approval.

#### 4.3.2 End-user empowerment

In Canada, it is important to the government that farmers continue to have a choice in selecting the most appropriate agricultural practices and products, including products developed using GEd techniques, and to choose those that offer the greatest economic, social, ethical, and environmental benefits. At the same time, empowering farmers to choose the method of production that best suits their needs, including access to safe applications of biotechnology, is important to enable their role as stewards of the land and ensure that they remain competitive in domestic and global markets.

#### 4.3.3 Global co-operation

Global cooperation in establishing regulatory frameworks for the application of GEd is much needed. This is because any potential unintended GEd-derived crisis will not be limited to a specific geographical region. Therefore, honesty and transparency of all parties involved, including governments, scientific research institutes, industrial entities, regulatory agencies, and inter-countries’ communication entities, will give clarity about the current GEd status. This should be performed to facilitate the safe and beneficial use of GEd to help in solving current challenges with respect to food security and climate change.

## 5 Discussion

Countries have approached regulation of GEd differently depending on their individual contexts, as seen in the results of our questionnaire. This is in line with the findings of Ishii and Araki (2017), who suggested that countries would likely be divided in their policies regarding GEd. For instance, those that embraced the commercialization of GM technology, such as Argentina, have been amongst the most proactive in adopting frameworks to include regulation of NBTs and their products. As a result, in 2015, Argentina developed a regulation, Resolution no. 173/15 of the Secretariat of Agriculture, Livestock and Fisheries, that incorporates the criteria and establishes procedures to determine in which cases a crop or animal obtained by breeding techniques involving modern biotechnology does not fall under GMO regulations (Whelan and Lema, 2015). In contrast, Canada which also plants and trades in GM products globally chose not to develop any new regulations for GEd products. Their product-based approach applies to GEd products in the same way as it does to products of conventional breeding, mutagenesis, and genetic engineering. Although the United States follows a similar product-based approach as Canada for GMO regulation, the three federal agencies with oversight of biotechnology each chose to handle GEd products differently.

Other countries have preferred to amend current regulations or issue guidance regarding how NBTs such as GEd will be handled. Australia, Japan, and two US federal agencies, the US-EPA and FDA, took this approach. Their guidance indicates that based on the kind of site-directed nuclease (SDN) technique applied, the final product may or may not be treated as a GMO. Whelan and Lema (2015) argue that new regulations should not be based on a closed list or description of particular technologies but rather should be framed to be flexible and applicable to existing and future technologies. This would indeed save the time and effort required to update or develop new regulations every time a new technology is discovered. In addition, regulations for existing technologies ought to evolve as we learn more about them.

GM regulation is very expensive because many of the regulatory studies, in effect, assess the potential for unexpected effects of the process in addition to unintended effects of the intended modification. These require exhaustive profiling studies that catalog differences from a control—the applicant then has to argue whether any of those changes are important. Regulatory studies are more meaningful if the regulatory authority defines what is regarded as significant and potentially harmful changes, and the applicant conducts studies that address those changes. By implementing the innovation principle, regulators could also define potentially beneficial changes; this would provide a good balance by not focusing only on potential risks but also weighing them against benefits when arriving at a decision that would be in the national interest (Raybould, 2021).

In the EU, a decision was made in 2018 by the ECJ indicating that products made from GEd would be treated in the same manner as GMOs. However, in November 2019, the European Council of Ministers asked the European Commission (EC) to conduct a study on the current regulatory framework for GMOs and determine whether it was sufficient to cover NBTs or whether there would be a need to revise the current regulations to address the new technologies. The results of the study released on 29 April 2021 revealed that NBTs have the potential to contribute to a more sustainable food system as part of the objectives of the European Green Deal and the Farm to Fork strategy. Furthermore, the study found that the current framework was not fit for its purpose, and the EC initiated a wide and open consultation process to discuss the design of a new legal framework for NBTs (https://ec.europa.eu/info/law/better-regulation/have-your-say/initiatives/13119-Legislation-for-plants-produced-by-certain-new-genomic-techniques_en).

Responses from the regulatory officers and academics showed that GEd applications submitted to countries so far are diverse in terms of species and traits comprising disease resistance, welfare, and bio-fortification. This pattern is in line with previous studies where it was noted that in selecting which applications to focus on, many of the developers of GEd organisms have sought to learn from the controversies surrounding GM crops, as well as welfare and ethical issues particular to animals (Bruce, 2017). Furthermore, many of the traits in the applications could potentially contribute to sustainable agricultural development by addressing issues such as food security (yield improvement), malnutrition (biofortification), and climate change adaptation (rain, salinity, drought, and heat tolerance). These observations have also been made in recent studies conducted looking at how GEd crops and livestock can contribute toward food security and climate change adaptation (Karavolias et al., 2021; Smyth et al., 2021).

The advent of new technology brings with it challenges and opportunities. For the selected countries, there were many similarities, irrespective of the region. Some of the common themes that emerged with regard to challenges include concerns about international trade due to regulatory asymmetry, public perception, and acceptance of the GEd products and potentially detrimental effects. With regard to opportunities, many acknowledged that GEd provides a diverse toolbox that can contribute to sustainable agriculture by producing climate-resilient crops and livestock. Additionally, due to the accessibility and affordability of GEd technology, there is equitable access to a wide range of players, especially for small-scale companies and public institutions with limited budgets (Lema, 2019).

Some scientists have proposed harmonization of regulations for GEd to avoid the experience of GM technology and its products. This might prove a challenge based on the different legal definitions adopted by countries and individual contexts. Rather, countries can have discussions and seek consensus on issues that cut across regulatory regimes such as risk assessment (RA) data requirements and mutual recognition of other countries’ RA data when it comes to international trade and agriculture. As part of the regulatory process, both environmental and food safety assessments are carried out for GM crops and animals. In most countries, currently, policy protection goals set in the regulations are often too broad and ambiguous, leaving risk assessors to interpret them without guidance on what effects are considered detrimental, resulting in concerns about accountability, transparency, and consistency of the RA process (Garcia-Alonso and Raybould, 2014). From the questionnaire and interview responses received, countries indicated that there are no unique data requirements for GEd crops and animals, and they will undergo the same RA as GMOs. Not designing new or revising existing RA frameworks for GEd crops and animals, whether a product or process-based regime is used, might create the impression to technology developers and scientists that they are no different from GMOs and hence will be subjected to the same regulatory process. If countries want to encourage innovation and uptake of new technologies such as GEd, then they have to adapt to their regulatory systems accordingly and not restrict this to the legal classification of products from GEd as GMOs or not. Ideally, countries would agree on a common framework for RA, which can then be adopted to suit local frameworks by individual countries.

A second challenge raised by questionnaire respondents was that of public perception and acceptance of GEd products. According to Malyska et al. (2016), approval of products of certain technology by a regulatory agency does not guarantee acceptance by society. During public participation forums, regulators often seek to assure the stakeholders present that a product that has received regulatory approval is deemed safe for public consumption**.** Although people may agree with this, they might be concerned about other aspects of farming and agriculture in general that regulators may not sufficiently address. Therefore, emphasizing that products meet regulatory standards may not be a sufficient answer to those opposing a technology and consequently could lead to more restrictions as regulation tries to catch up with public perception (Malyska et al., 2016). This brings about the aspect of communication that will be a key factor in the acceptance of products of GEd.

Knowlton (2017) explains that designing optimistic messages in relation to new technologies often improves their acceptance by the public. The author found that optimistic messages inspire and energize people to find solutions to problems (Knowlton, 2017). Often when trying to advocate for solutions to food insecurity, messages crafted seem to imply that if a certain technology is not adopted, millions of people around the world face starvation (Raybould A., 2019). Although food insecurity is a serious threat, presenting messages that advocate for better farming practices and using technology as one of the tools to address this problem might be a better approach than giving alarming scenarios in case a technology is not accepted. The challenge presented to technology developers will, therefore, be to create optimistic messages about the traits or the challenges a GEd crop or animal will address. In conclusion, although establishing regulatory frameworks that support the uptake of innovations is important, empowering the users of the technology is also necessary. Empowering farmers and technology end users to make the decisions that best suit their conditions or day-to-day livelihoods will contribute to greater acceptance and adoption of innovations.

## 6 Conclusion

This article gives a picture of the early responses by selected countries to products of GEd technology. Some have chosen to amend their current regulations to accommodate GEd, while others handle them on a case-by-case basis, having developed new regulations to cater to this new technology. In contrast, some have chosen to apply existing GMO regulatory frameworks for oversight of GEd products. Depending on the approach taken, it remains to be seen how stakeholders in the individual countries will respond. Also, despite different approaches to regulation, global consensus and harmonization for issues that cut across countries will be important to avoid regulatory bottlenecks and disruptions to global trade.

Based on the applications submitted to regulatory authorities, it is evident that the traits targeted in both crops and animals have the potential to contribute toward sustainable agricultural development. A key component for them to achieve this will be enabling regulatory frameworks that will provide a path to market for each of these products. This is particularly important for countries where food security remains a challenge and is made worse by climate change effects such as drought and floods, such as in sub-Saharan Africa.

GEd technology presents both opportunities and challenges, and it will be up to countries to decide how to best exploit the opportunities and handle challenges as the technology develops. Factors that will be important are regulatory certainty which reassures developers and innovators to invest in the technology and effective communication and transparency to the public, and stewardship for end users such as the farmers to empower them to make the best decision for their livelihoods (Bailey-Serres et al., 2019; Chataway et al., 2006).
