# Supplementary material for: Regulatory frameworks can facilitate or hinder the potential for genome editing to contribute to sustainable agricultural development
Source: Front Bioeng Biotechnol. 2022 Sep 30;10:959236. doi: 10.3389/fbioe.2022.959236 (PMC9562833; doi:10.3389/fbioe.2022.959236)
Supplement: Supplementary file 1 [file Table1.DOCX]

**Gene editing regulatory review questions**

*The below series of questions seeks to find out how countries currently handle applications involving gene edited organisms. With this questionnaire, you, as a regulatory expert from X country, are kindly asked to contribute by answering the following questions.*

*You are requested to describe your country situation as accurately as possible in each of the questions. However, the answers can be based on your expert opinions, and do not necessarily have to reflect the views and positions of your institution. The responses provided will be used to write a paper regarding the current regulatory regimes for genome edited organisms.*

1. Please state the name of your country and Institution you work for.
2. What role do you currently play in your institution?
3. What is the regulatory trigger for genetically engineered/modified organisms in your country?
4. Product-based
5. Process-based
6. Please indicate the option that best describes regulation of genetic engineering technology in your country?
7. Only one National agency is responsible for oversight in my country (Please indicate the name)
8. One agency is responsible for oversight but works in partnership with other government agencies in my country
9. Multiple government agencies are responsible for oversight in my country
10. Not applicable
11. Other
12. If your answer is b and c in question 4, please list the organisations involved in the regulation/oversight of genetic engineering technology. How are responsibilities divided among the partner organisations?
13. Has your country received an application involving new breeding techniques such as gene editing? If yes, how many, date when received?
14. Yes
15. No
16. If indicated yes above, what has been the nature of applications received so far, for research purposes, placing on the market/commercial release? What was the organism involved (plant, animal, microorganism)?
17. Please select the option that best describes how your country currently handles applications involving Genome Edited (GE) organisms
18. Current regulations were amended to include new breeding techniques
19. Gene editing applications are handled on a case-by case basis
20. New regulations were developed to cater for new breeding techniques e.g. gene editing
21. No new regulations were developed and GE organisms are subjected to same oversight as their conventional counterparts
22. Not applicable
23. What are the decision-making criteria for applications involving gene edited organisms? Do they differ from that of GMOS?
24. Are there separate guidelines/regulations for GE crops and animals?
25. What kind of risk assessment data do you require for applications involving gene edited organisms? Does this differ from that of genetically modified organisms?
26. Is there any stakeholder engagement done during the regulatory process in your country?
27. Yes
28. No
29. If your answer is (a) above, please indicate the option that best describes stakeholder engagement in your country
30. Stakeholders are engaged at the beginning of development of regulations/guidelines
31. Stakeholders are engaged after a draft regulation/guideline is developed and their input/comments are sought
32. Stakeholders are never engaged during development of regulations/guidelines
33. Other
34. If your answer is (a) above, please expound on how the stakeholder engagement is used to develop policy and inform decision-making
35. In your country context, what are the potential challenges of gene edited organisms?
36. In your country context, what are the potential benefits/opportunities of gene edited organisms?
37. In your country context, what factors do you think are key to create an enabling environment for exploitation of new technologies such as gene editing to solve various challenges in your country?
38. Is your country a signatory to any international agreement, for example, The Cartagena Protocol on Biosafety?
39. How has this affected the establishment of the regulatory framework in your country?
