# Supplementary material for: Regulatory frameworks can facilitate or hinder the potential for genome editing to contribute to sustainable agricultural development
Source: Front Bioeng Biotechnol. 2022 Sep 30;10:959236. doi: 10.3389/fbioe.2022.959236 (PMC9562833; doi:10.3389/fbioe.2022.959236)
Supplement: Supplementary file 2 [file Table2.DOCX]

**Supplementary information on countries and institutions where questionnaire interview respondents came from**

| **Country** | **Institution** |
| --- | --- |
| Argentina | National University of Quilmes; Bernal, Argentina |
| Australia | Office of the Gene Technology Regulator |
| Brazil | National Technical Committee on Biosafety, CTNBio, from the Ministry of Science, Technology and Innovation of Brazil |
| Canada | Agriculture and Agri-Food Canada |
| China | Northwest A&F University Yangling, China |
| EU | Federal Republic of Germany, Federal Office of Consumer Protection and Food Safety (BVL) |
| Japan | Nagoya University Japan |
| Kenya | National Biosafety Authority |
| USA | United States Department of Agriculture(USDA) |
| Zambia | National Biosafety Authority |
