# Supplementary material for: Regulatory frameworks can facilitate or hinder the potential for genome editing to contribute to sustainable agricultural development
Source: Front Bioeng Biotechnol. 2022 Sep 30;10:959236. doi: 10.3389/fbioe.2022.959236 (PMC9562833; doi:10.3389/fbioe.2022.959236)
Supplement: Supplementary file 3 [file Table3.DOCX]

**Specifications of the GEd regulation approach in USA**

| **Country** | **GEd regulatory approach** |
| --- | --- |
| USA | **Depends on the agency and situation.**   1. **Current regulations were amended to include new breeding techniques**   Food and Drug Administration (FDA)/ Center for Veterinary Medicine (CVM) – a draft update to Guidance for Industry (GFI) #187 was released in 2017. The original final 2009 guidance was for the “Regulation of Genetically Engineered Animals Containing Heritable Recombinant DNA Constructs.” The draft updated GFI#187 addressed animals whose genomes have been intentionally altered using modern molecular technologies, including genome editing.  Environmental Protection Agency (EPA) – published a proposed a new rule in 2020, proposing to exempt from an existing mandatory licensing process certain PIPs created via biotechnology. The proposed exemption is for Plant Incorporated Protectants (PIPs) created through gene editing which could be achieved through conventional breeding with sexually compatible plants.   1. **Gene editing applications are handled on a case-by case basis**   FDA/CVM - evaluates products using a risk-based approach and on a case-by-case basis. In cases where this risk is determined to be low, FDA may not expect developers to submit information to them at all (e.g., Intentional Genomic Alterations (IGAs) used in non-food research animals, such as mice). In some cases, FDA reviews data and information submitted prior to marketing by developers to support that the product is low risk, and if FDA determines the product is in fact low risk it does not enforce the approval requirements in these cases (e.g. IGAs used in disease models in animals of food-producing species used in a research setting). Where the product is not low risk, FDA expects submission of an approval application.   1. **New regulations were developed to cater for new breeding techniques e.g. gene editing**   United States Department of Agriculture (USDA) – new Sustainable, Ecological, Consistent, Uniform, Responsible, Efficient (SECURE) rule (2020), updates USDA’s regulatory approach. The SECURE rule applies to any organism developed using genetic engineering that is or may pose a plant pest risk. The rule expands the definition of genetic engineering beyond the use of rDNA to include the use of nucleic acids (not just DNA) that have been synthesized or amplified to modify or create a genome. The Animal and Plant Health Inspectorate Service (APHIS) SECURE rule applies to organisms modified using genome editing as well as rDNA technologies.  In 2017, FDA issued a Request For Information (RFI) on foods from genome edited plant varieties (https://www.federalregister.gov/documents/2017/01/19/2017-00840/genome-editing-in-new-plant-varieties-used-for-foods-request-for-comments). FDA intends to develop draft guidance for industry based on the information gleaned from the RFI and is continuing to work to produce such guidance. At the same time, FDA explained in the RFI that it would accept voluntary premarket consultations on food from genome edited plant varieties. |
